# The article ‘Sex, gender and gender identity’ fails to adequately engage with the extant scientific literature

**DOI:** 10.1192/bjb.2021.77

**Published:** 2021-10

**Authors:** Florence Ashley

**Affiliations:** University of Toronto, Canada Email: f.ashley@mail.utoronto.ca

The recent paper titled ‘Sex, gender and gender identity: a re-evaluation of the evidence’, which appeared as a Special Article in the *BJPsych Bulletin*, contains concerning misrepresentations of the scholarly literature. The article selectively cites sources to make claims that are contrary to the available literature and best practices, which strongly support access to social, legal and medical transition. Using a direct quote, the authors claim that a Royal College of Psychiatrists position statement includes ‘placing barriers [to] medical transition’ within the meaning of conversion therapy. However, the document actually says ‘place barriers in the way of trans people who seek advice regarding medical and/or social transition’.^1^ Raising barriers to transition and raising barriers to people seeking advice are two different things altogether, and the elision misrepresents the Royal College of Psychiatrists’ position statement. Substantively altering quotes in this way is contrary to ethical authorship practices and is difficult to explain. While I would agree that preventing transition is a form of conversion therapy, as expressed in a recent report by UN Independent Expert Victor Madrigal-Borloz,^2^ this is not what the cited position statement says.

Despite discussing these topics, the authors fail to cite available data on the prevalence of anti-trans conversion therapy in the UK;^3^ fail to acknowledge strong methodological criticisms of studies suggesting that most transgender youth grow up to be cisgender;^4,5^ fail to refer to empirical evidence of the harmfulness of misgendering and deadnaming;^6–9^ fail to mention that youth referred to gender identity clinics are significantly similar to those in the past despite changes in gender ratios;^10^ fail to substantially engage with significant evidence of mental health benefits to transition by brushing it off as immaterial to youth;^11^ fail to refer to evidence demonstrating that trans youth can be trusted about who they are and do not plausibly transition out of internalised homophobia;^12–15^ and fail to refer to a wealth of data showing transition and gender affirmation to have an important positive impact on mental health.^16–23^ Many references used in the article in support of substantial and contentious points are of poor quality and include news reports, non-peer-reviewed blog posts and a Tumblr survey.

Scientific literature must meet a high standard for publication. The editorial and peer-review process is intended to screen for problems such as these, which gravely threaten the quality of published works and undermine the public's ability to rely on scholarly publication as an indicium of reliability. Providing competent care for transgender youth depends upon the integrity of the scientific process, of which publication is an important part. The past few years have seen a concerning rise in articles opposing gender-affirming care being published in scientific journals despite failing to engage with the available literature and, at times, being directly contradicted by it. Transgender communities deserve high-quality research and scientific publications.

Gender-affirmative care remains the leading approach to care for transgender youth and is well supported by empirical evidence, clinical experience and ethical reasoning.^24,25^ Contrary to what the article's authors imply, gender-affirmative care does not foreclose youth's futures. Quite the contrary.^26^ Attempts to unduly delay or prevent social, legal or medical transition among youth who desire them should be opposed. Children and adolescents deserve unconditional acceptance and love at all points in their lives, including in their gender.
